# Combined cardiac surgery in a Marfan syndrome patient with severe scoliosis via lower hemisternotomy: a case report

**DOI:** 10.1186/s40792-022-01504-8

**Published:** 2022-07-27

**Authors:** Shun-Ichiro Sakamoto, Ryosuke Amitani, Yusuke Motoji, Takako Yamaguchi, Atsushi Hiromoto, Kenji Suzuki, Yosuke Ishii

**Affiliations:** 1 Department of Cardiovascular Surgery, Nippon Medical School Musashikosugi Hospital, 1-396 Kosugimachi, Nakahara-ku, Kawasaki-shi, Kanagawa 211-8533 Japan; 2 Department of Cardiovascular Surgery, Nippon Medical School, 1-1-5 Sendagi, Bunkyo-ku, Tokyo, 113-8603 Japan

**Keywords:** Marfan syndrome, Scoliosis, Cardiac surgery

## Abstract

**Background:**

Scoliosis is one of the symptoms manifested by patients with Marfan syndrome (MFS). Deformity of the thoracic cavity due to severe scoliosis may cause difficulty during cardiac surgery in terms of the surgical approach and instrument manipulation; however, only a few reports have been available regarding the surgical case of MFS with severe scoliosis. Here, we report a case of combined aortic valve replacement and left atrial appendage closure in a patient with MFS who had severe scoliosis using lower hemisternotomy.

**Case presentation:**

A 62-year-old female with MFS was referred to our hospital after being diagnosed with severe aortic regurgitation and paroxysmal atrial fibrillation with a history of cerebral thromboembolism. The aortic valve showed severe insufficiency due to cusp prolapse, whereas the aortic root was moderately dilated (42 mm). Echocardiography revealed severe regurgitation with reduced left ventricular ejection function (32%) and massive left ventricular diastolic dimension (88 mm). Moreover, combined aortic valve replacement and left atrial appendage closure was indicated. However, the patient had chest deformity due to severe scoliosis. Thus, conventional full sternotomy or thoracotomy was considered an inappropriate surgical approach. Lower hemisternotomy was selected on the basis of three-dimensional reconstruction imaging of the aorta, left atrial appendage, sternum, and rib. Sternal elevation and rib retraction with the costal arch folded back provided enough surgical field for the combined procedures to be safely conducted. The postoperative course was uneventful, except for predicted prolonged mechanical ventilation with the assistance of intraaortic balloon pumping. Thereafter, the patient has been free from any cardiac and cerebrovascular event.

**Conclusions:**

Lower hemisternotomy can be useful for combined cardiac surgery in MFS with severe scoliosis.

## Background

Scoliosis is a one of the symptoms manifested by patients with Marfan syndrome (MFS). Deformity of the thoracic cavity due to severe scoliosis may cause the difficulty in cardiac surgery in terms of surgical approach and instrument manipulation; however, only a handful of reports have been available regarding the surgical management of MFS with severe scoliosis [1, 2]. In this report, we present a case of combined aortic valve replacement and left atrial appendage closure in a patient with MFS who had severe scoliosis. Lower hemisternotomy can be considered the optimal surgical approach for successful treatment. Written informed consent for the publication of case details and images was obtained from the patient before drafting of the manuscript.

## Case presentation

**Fig. 1:**
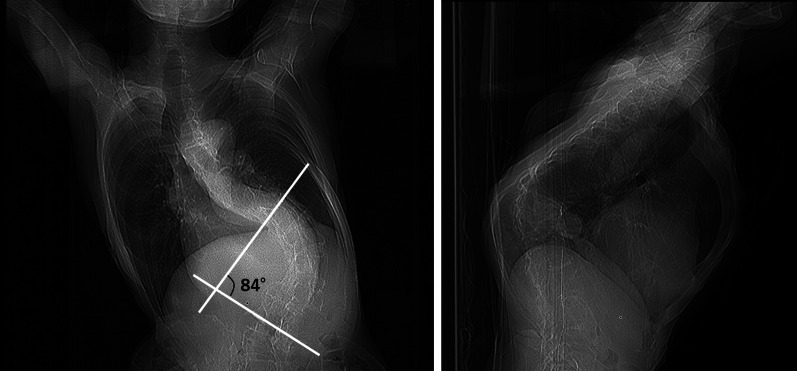
Postero-anterior and lateral chest X-ray showing severe scoliosis (Cobb angle measurement of 84 degrees)

**Fig. 2:**
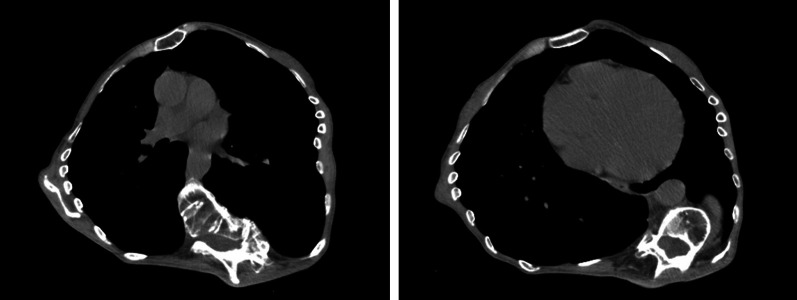
Preoperative chest computed tomography showing deformity of the pleural cavity and distortion of spine

**Fig. 3:**
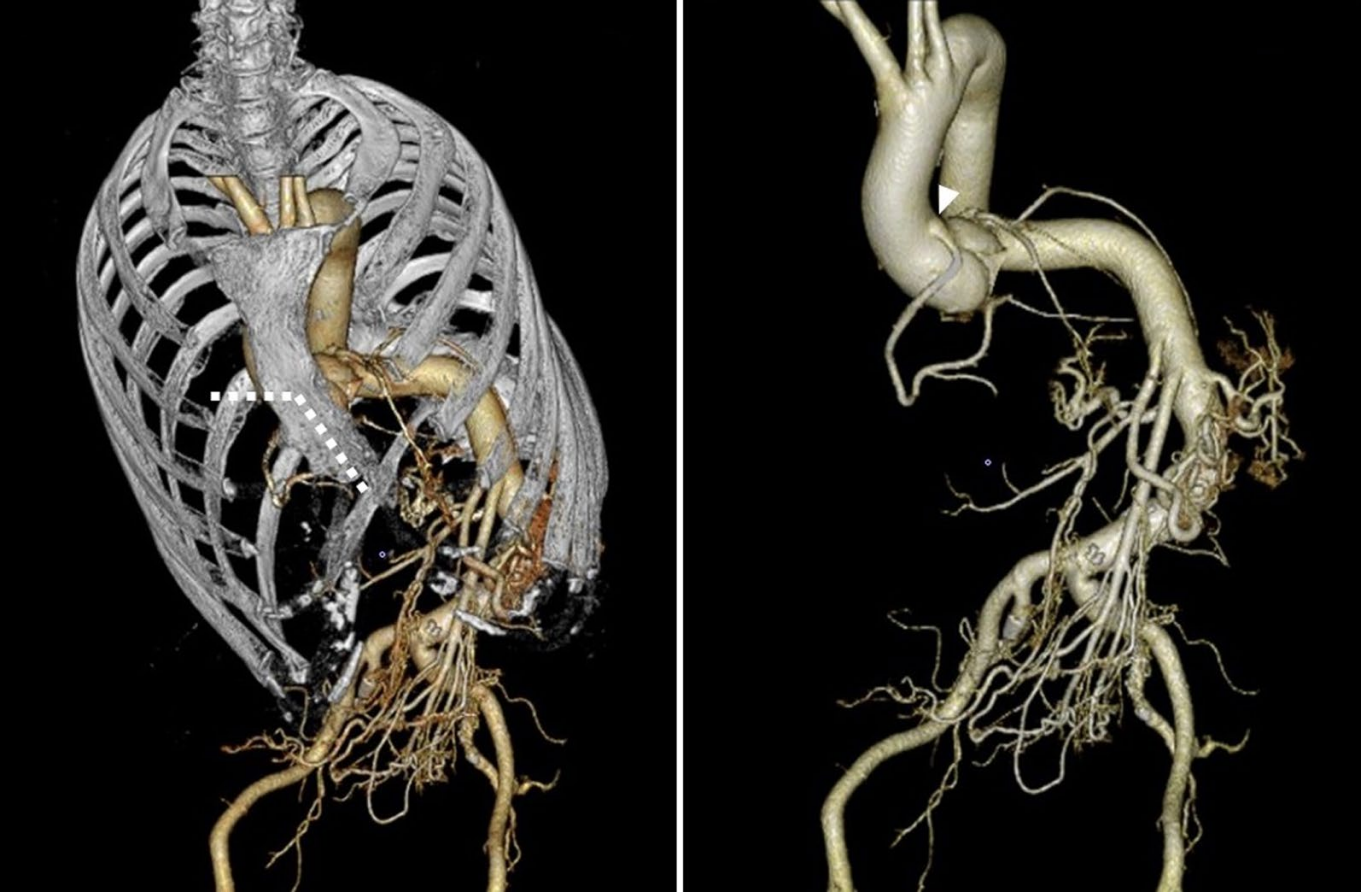
Three-dimensional reconstruction imaging showing the surgical incision line for lower hemisternotomy (white dotted line). The right coronary artery arises from the anteromedial surface of the ascending aorta (white arrowhead)

A 62-year-old female with MFS was presented to the hospital for an emergency after being diagnosed with cerebral thromboembolism due to atrial fibrillation (AF). Apart from the acute endovascular thrombectomy, the patient was treated for congestive heart failure associated with aortic valve regurgitation with tachycardic AF. Echocardiography revealed severe regurgitation with reduced left ventricular ejection function (32%) and massive left ventricular diastolic dimension (88 mm). Aortic valve was tricuspid, and the eccentric regurgitation jet revealed prolapse of right coronary cusp. The aortic root was moderately dilated (42 mm). After 2 months of treatment, the patient was referred to our hospital for the surgical treatment of aortic valve regurgitation. The patient was free from the sequela of brain stroke, but complained of general fatigue consistent with a New York Heart Association class III condition with weakened leg muscle strength due to disuse syndrome. N-terminal pro-brain natriuretic peptide (NT-proBNP) level at admission was 3964 pg/mL. Sinus rhythm had been maintained after initiating oral amiodarone. The diameter of the aortic root in this patient was below the size required for indicating surgical treatment for MFS [3]. In addition, the dimension and systolic function of the left ventricle suggested the progression of ventricular remodeling. Therefore, aortic valve replacement was selected primarily for the prevention of heart failure. In contrast, there were various options for the management of AF in the present case. Left atrial appendage closure was thought to be an appropriate treatment option to prevent recurrence of cerebral thromboembolism because AF had been effectively controlled by antiarrhythmics and the patient would receive coumadin following mechanical valve implantation. Thus, combined surgery involving aortic valve replacement and left atrial appendage (LAA) closure was planned in order to achieve early rehabilitation and return to daily life. Her chest XP revealed severe scoliosis (Fig. 1). Spirometry showed normal lung function. A computed tomography was performed to determine the optimal intercostal space to access both the aortic valve and LAA (Fig. 2). Three-dimensional reconstruction imaging showed that right lower hemisternotomy in a reverse “L” fashion was the optimal approach for clamping and incising the aorta with the ectopic origin of the right coronary artery arising from anteromedial surface of ascending aorta (Fig. 3).

**Fig. 4:**
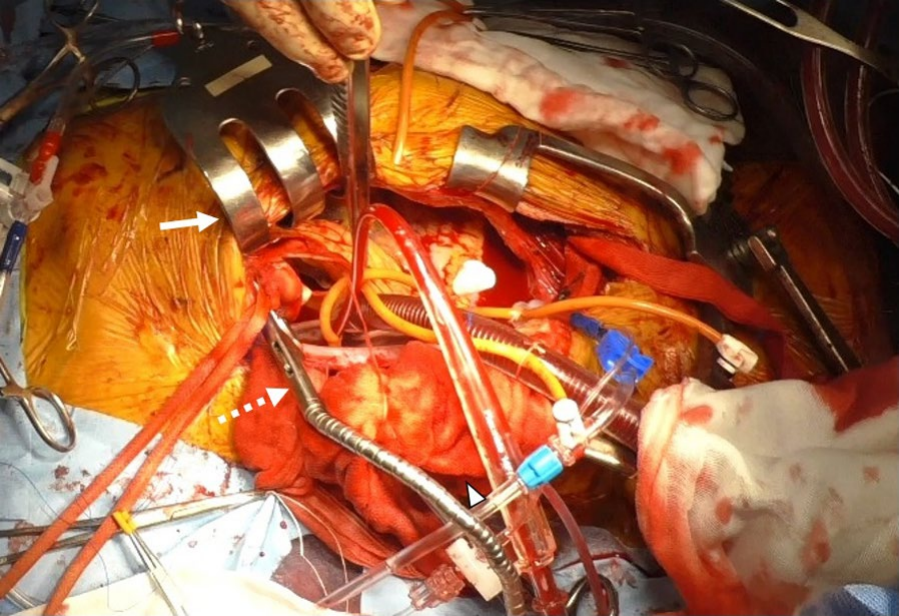
Securing the operating field. The aorta was cross-clamped using a flexible aortic clamp (white dotted arrow). A Kent retractor was applied to elevate the sternum (white arrow). The right costal arch was folded back (white arrowhead) and fixed using the retractor

The patient was intubated using a double-lumen tube and laterally positioned at approximately 30 degrees right-side up. A skin incision was made from the second intercostal space down to the xiphoid process, after which the chest was opened via right lower hemisternotomy. The surgical field was secured using a Kent retractor (Takasago medical Inc., Tokyo, Japan) and sternal retractor with the costal arch folded back. Cardiopulmonary bypass was established with femoral arterial and cavoatrial cannulation. The ascending aorta was cross-clamped using flexible aortic clamp (Fig. 4). Cardiac arrest was obtained using bidirectional cardioplegia. An oblique aortotomy was created 10 mm medial to the ostium of the right coronary artery. The aortic valve cusp was then resected, and a 25-mm mechanical valve was implanted in the intra-annular position. After closing the aortotomy, the 40-mm AtriClip (Atricure Inc, Westchester, OH, USA) was applied to the stump of the LAA before releasing the aortic cross-clamp. The patient was carefully weaned from cardiopulmonary bypass, which required an intraaortic balloon pump for the treatment of perioperative low-output syndrome due to reduced left ventricular function. The extracorporeal circulation time and aortic cross-clamp time were 165 and 87 min, respectively. The total operative time was 303 min. Transfusion of 4 units of red blood cells and 6 units of fresh frozen plasma was required during the surgery.

The patient was extubated on postoperative day 2, and the intraaortic balloon pump was removed 2 days after the extubation. Further, she was then transferred to the rehabilitation center for the management of her disuse syndrome on postoperative day 21; thereafter, she has been living independently without any cardiac and cerebrovascular event.

## Discussion

MFS is an inheritable connective tissue disorder caused by mutations in the FBN1 gene. Studies have estimated its prevalence to be around 2–3/100,000 individuals, with a variety of manifestations in the ocular, skeletal, and cardiovascular systems [4]. In the present case, the patient’s disorder had been complicated with aortic valve insufficiency and severe scoliosis. Scoliosis occurs in approximately 62% of patients with MFS [5]. Reports have shown that thoracic deformity associated with scoliosis causes respiratory dysfunction due to the disturbance caused by the expansion of thoracic cavity with limited intercostal muscle function. Furthermore, according to the Cobb angle, which is quantified to evaluate the severity of scoliosis, the current patient requires surgical intervention of scoliosis [6]. Although the patient’s respiratory function was normal, prolonged mechanical ventilation was postoperatively predicted.

Cardiac surgery for MFS patients with severe scoliosis can be challenging given the difficulty in determining the appropriate surgical approach for the distorted heart axis. Although studies on mitral valve repair via thoracotomy have been reported, little is known regarding combined cardiac surgery [2]. The current patient required both aortic valve replacement and LAA closure. We expected that median sternotomy would be difficult during aortotomy and that right thoracotomy was not the appropriate approach to the LAA. Therefore, we opted for lower hemisternotomy to safely perform combined cardiac surgery.

Upper hemisternotomy can be useful during cardiac surgery, especially for the treatment of aortic valve surgery [7]. In the current case, lower hemisternotomy was selected since the heart had rotated counter clockwise and the aortic root was positioned lower than usual. In addition, we expected that sternal resection would be extended toward the 2nd intercostal space, requiring thoracotomy in case of an insufficient surgical field for approaching the LAA. Any procedure that required a thoracotomy could be handled effectively using a double-lumen tube. However, sternal elevation using a Kent retractor was helpful in obtaining sufficient space for aortic clamping and delivery of the device for LAA closure. Furthermore, the divided sternum was folded back at the costal arch resembling an open door, which allowed us to better secure the surgical field.

Immature collagen matrix in the rib cartilages has been suggested to cause various pectus deformities, such as pectus excavatum and pectus carinatum, in patients with MFS [8]. Our case presented with pectus carinatum as well as severe scoliosis. Softening of the costal arch due to abnormal growth or elongation in MFS might allow it to be folded back. Surprisingly, the patient had little pain in this area after surgery. Our experience suggested that lower hemisternotomy with the costal arch folded back was less invasive, although it remains to be determined whether this approach can be used in all patients with MFS.

## Conclusions

Lower half sternotomy can be useful for combined cardiac surgery in MFS with severe scoliosis.

## Data Availability

Data sharing not applicable to this article as no datasets were generated or analyzed during the current study.

## Abbreviations

MFS: Marfan syndrome
AF: Atrial fibrillation
LAA: Left atrial appendage
